# Spectrofluorimetric and Spectrophotometric Determination of Pregabalin in Capsules and Urine Samples

**Published:** 2010-09

**Authors:** Rasha Abdel-Aziz Shaalan

**Affiliations:** *Pharmaceutical Analytical Chemistry Department, Faculty of Pharmacy, Alexandria University, Elmessalah 21521, Alexandria, Egypt*

**Keywords:** pregabalin, fluorescamine, 2,4-dinitroflurobenzene, chloranil, urine

## Abstract

Three new, simple, sensitive and selective spectrofluorimetric and spectrophotometric methods were developed for the determination of the γ-amino-n-butyric acid derivative, pregabalin. Pregabalin as a primary amine reacts with fluorescamine to yield a fluorescent product (Method I), with 2,4-dinitrofluorobenzene (Method II) and 2,3,5,6-tetrachloro-1,4-benzoquinone (Method III) in aqueous alkaline buffered media to form colored products which could be measured spectrophotometrically. The optimum conditions for each reaction were ascertained and the methods were applied for the determination of pregabalin over the concentration range of 20-280 ng mL^-1^ and 1-7 μg mL^-1^ for spectrofluorimetry and spectrophotometry, respectively with good correlation (≥0.999). The limits of assays detection ranged from 9.6 × 10^-4^ μg mL^-1^ to 0.42 μg mL^-1^ for spectrofluorimetry and spectrophotometry, respectively. The suggested methods were applied to the determination of the drug in capsules. No interference could be observed from the additives listed to be in capsules. Furthermore, the spectrofluorimetric method was extended to the in-vitro determination of pregabalin in spiked urine, interference from endogenous amino acids could be eliminated through selective complexation with copper acetate; the percentage recovery was found to be 98% ± 1.42 (n=6). Co- administered drugs such as chlordiazepoxide, clonazepam, diazepam, nitrazepam and lamotrigine did not interfere with the assay. The methods were validated with respect to linearity, accuracy, precision and robustness. The results obtained were determined to be in good agreement with those obtained using a previously reported method.

## INTRODUCTION

Pregabalin (PRG), (S)-3-(aminomethyl)-5-methylhexanoic acid, is an antiepileptic used as an adjunct in the treatment of partial seizures. It is also used in the treatment of generalized anxiety disorder, neuropathic pain and fibromyalgia (1).

PRG is not yet official in any pharmacopeia neither in USP 2007 nor BP 2010. The reports found in the literature for PRG determination concentrate on chromatographic methods, these include: HPLC either using atmospheric pressure chemical ionization tandem mass spectrometric method (2), precolumn derivatization with either picryl sulphonic acid (3), or o-phthalaldehyde associated with fluorescence detection (4, 5). Enantiospecific analysis of PRG was carried out through HPLC-ELSD (6), and chiral precolumn derivatization (7, 8). Separation and characterization of modified pregabalins in terms of cyclodextrin complexation, was reported using capillary electrophoresis and nuclear magnetic resonance (9). Only four reports were found for the spectrophotometric and spectrofluorimetric determination of the drug in bulk powder and pharmaceutical preparations; these are UV spectrophotometry where the A was measured at 210 nm (10), study of the charge transfer complexes of PRG with several π- acceptors (11, 12), reaction of PRG with 7-chloro-4-nitrobenzofurazon (NBD-Cl) for spectrophotometric and spectrofluorimetric determination of the drug (13). Among these spectroscopic reports, only the UV method (10) was extended for the determination of PRG in spiked urine samples.

Since spectrofluorimetry and spectrophotometry are the technique of choice in research laboratories, hospitals and pharmaceutical industries due to its low cost and inherent simplicity, the present work was aiming to develop new simple, sensitive and selective spectrofluorimetric and spectrophotometric procedures for determination of PRG in dosage forms and spiked urine samples. The proposed spectrophotometric method (III) has distinct advantage over the reported method (12) regarding simplicity, sensitivity and limit of detection. Furthermore the proposed spectrofluorimetric method using fluorescamine is simpler and more sensitive than the reported fluorimetric method (13).

## EXPERIMENTAL PROCEDURE

### Instrumentation

- A Shimadzu spectrofluorophotometer model RF-1501 version 3.0 (Kyoto, Japan) using 150 W Xenon lamp and 1- cm quartz cell.
- Specord S600 spectrophotometer, associated with WinAspect software version 2.3, Analytik Jena AG, Germany.
- Digital pH meter 3310 Jenway.
- Thermostated water bath (Köttermann Hänigsen, Germany).

### Materials and Reagents

All chemicals and solvents used through this study were of analytical grade.

- Fluorescamine was purchased from Aldrich Chemical Co. Ltd. Gillingham Dorest- England. A solution containing 0.2 mg mL^-1^ was prepared in acetone.
- 2,4- Dinitrofluorobenzene, Hopkin and Williams Co., Essex-UK was prepared as 5 mg mL^-1^ in methanol. The solution should be protected from light and prepared fresh daily.
- Chloranil BDH Chem. Ltd. Poole, England was prepared as 0.246 mg mL^-1^ solution in ethanol.
- Borate buffer solutions of pH 8.5, 8 and 9.2 were used.
- Copper acetate, Chemajet Chem. Co., Egypt: 70 mg mL^-1^ aqueous solution was used.
- Pregabalin was donated from European Egyptian Pharm. IND. Alexandria- Egypt.
- Capsules were purchased from the local market Lyrica® capsules (Pfizer Egypt, S.A.E. Cairo, A.R.E. under authority Pfizer Inc. U.S.A.) labeled to contain 150 mg PRG per capsule, (Batch No. 0217107, Manufacturing date 10/2007, Expiry date 9/2010).
- Urine samples were collected from healthy volunteers.

The reagent solutions were stable for at least one week if kept in the refrigerator.

### Preparation of the standard solution

Stock solution of PRG was prepared as 0.1 mg mL^-1^ in water. For spectrofluorimetry a working solution was prepared as 4 × 10^-3^ mg mL^-1^ in the same solvent. The solutions were stable for one week if kept in the refrigerator.

### Preparation of capsule sample solution

The contents of twenty capsules were weighed and mixed. A quantity of the powder equivalent to 10 mg of PRG was transferred into a 100 mL volumetric flask, dissolved in water, and sonicated for 5 min., the volume was then completed with water, shaken well for 5 min. and filtered into a dry flask. Aliquots of the filtrate were diluted quantitatively to suit the linearity range of each particular assay method.

### Construction of calibration graphs

**Method I.** To a set of 10 mL volumetric flasks, appropriate aliquots of the standard working solution were accurately transferred. To each flask, 1 mL borate buffer solution of pH8.5 followed by 1 mL fluorescamine solution were added, the solutions were left for 25 min. at room temperature, then were made to volume with water and mixed well. The resulting fluorescence was measured at 485 nm using λ_ex_ 385 nm. The relative fluorescence was plotted versus the final concentration. Alternatively, the corresponding regression equation was derived.

**Methods II and III.** Different aliquots of the standard stock solution (Table 1) were transferred into two sets of 10-mL calibrated flasks. The specified volume of borate buffer of appropriate pH and reagent solutions were added (Table 1), the flasks were heated on a water bath (the temperature and time of heating were cited in table 1) and then cooled to room temperature. The volumes were made to the mark with distilled water and the absorbances were measured at wavelengths of maximum absorption against reagent blanks. For method II the solutions were neutralized with 5 M HCl before completion to the volume. The absorbances were plotted against concentration to construct the calibration curves, and the regression equations were computed.

**Table 1:** Assay parameters for the determination of pregabalin using the proposed methods

Item	Method		
	I	II	III

Standard conc. (mg mL^-1^)	4 × 10^-3^	0.1	0.1
Volume of standard solution (mL)	0.05-0.7	0.2-0.7	0.1-0.7
Borate buffer pH	8.5	8	9.2
Borate buffer volume (mL)	1	1	2.5
Reagent conc. (mg mL^-1^)	0.2	5	0.246
Reagent volume (mL)	1	0.8	3
Heating temp (°C)	R.T.	80	70
Heating time (min)	25	15	30
5M HCl volume (mL)	-	0.1	-
λ_max_ (nm)	Ex385	369	352
	Em485		

### Analysis of capsules

Different aliquots from the capsule sample solution were analyzed as under construction of calibration graphs. The nominal content of the capsules were calculated using the regression equation appropriate for each particular assay method.

### Analysis of urine samples using fluorescamine

Different aliquots of spiked urine were transferred into Pyrex test tubes. 1 mL copper acetate solution was added and the test tubes were heated in a boiling water bath for 30 min. The solutions were filtered into 10 mL volumetric flasks. The test tubes and the filter paper were washed with 2 mL of water; the washings were passed into the same flasks. The procedure was completed as under method I. The concentration of the drug was determined from the corresponding regression equation.

## RESULTS AND DISCUSSION

PRG, like other antiepileptic drugs that are structurally related to the neurotransmitter γ-amino-n-butyric acid, exhibits very low UV absorption (14), hence conventional UV spectrophotometric methods are of poor sensitivity. The therapeutic importance of PRG was behind the development of new and more sensitive spectrofluorimetric and spectrophotometric methods for its determination.

### Method I

**Figure 1:**
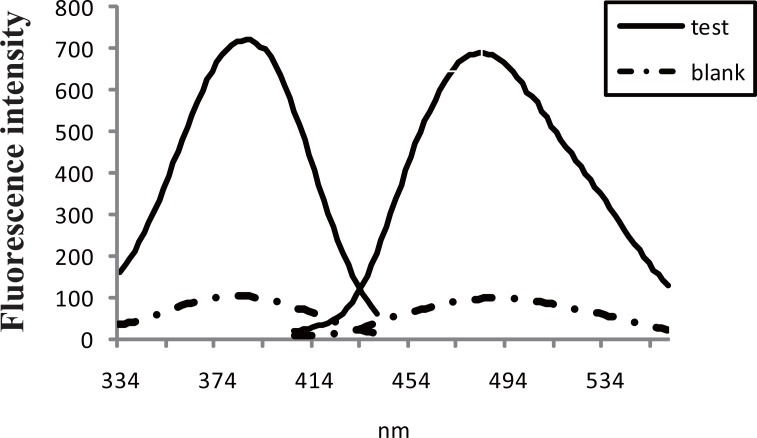
Fluorescence spectrum of the reaction product of 200 ng mL^-1^ pregabalin with fluorescamine.

**Figure 2:**
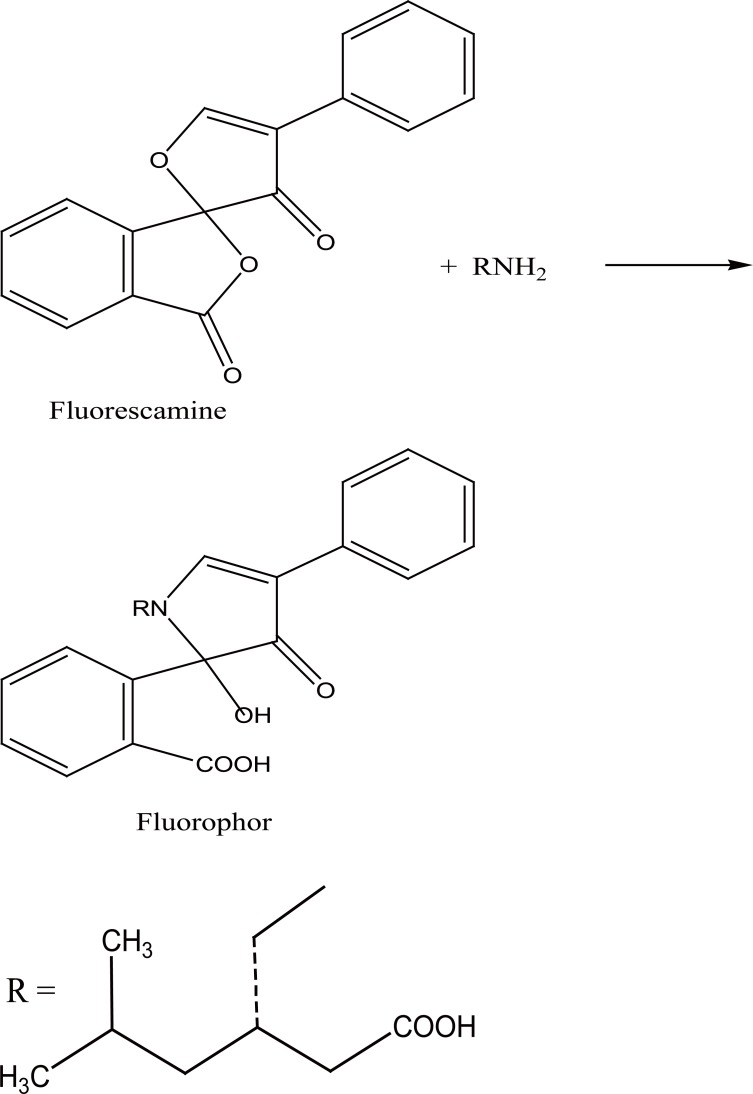
Proposal of the reaction pathway between pregabalin and fluorescamine.

PRG contains a primary aliphatic amino group which reacts at room temperature with fluorescamine in alkaline buffered solution, to yield a highly fluorescent yellow adduct (Fig. 1). The reaction mechanism is proposed to proceed as Figure 2 (14).

**Figure 3:**
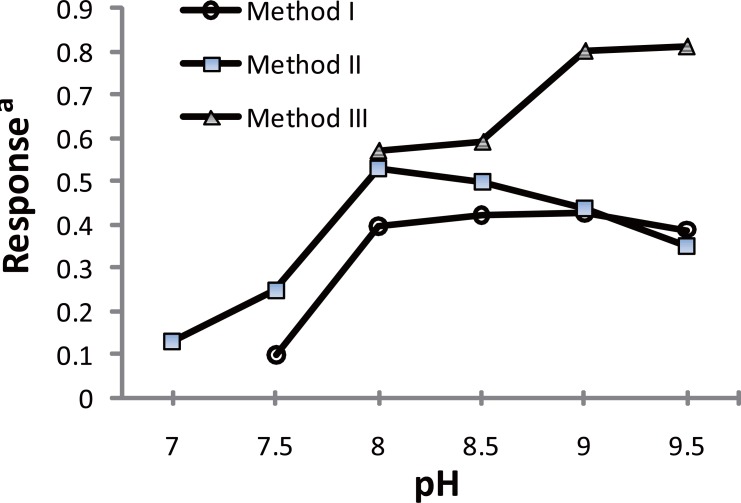
Effect of buffer pH on the reaction of PRG with the specific reagent. ^a^response is the A_max_ or the relative fluorescence × 10^3^.

**Figure 4:**
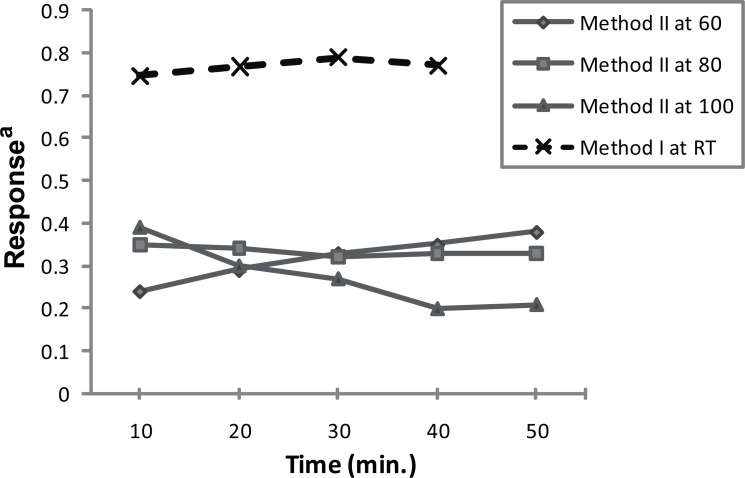
Effect of reaction time at room temperature on the reaction of PRG (0.25 μg mL^-1^) with fluorescamine. Effect of heating temperature and time on the substitution reaction of PRG(5μg mL^-1^) with 2,4 DNFB. ^a^response is the A_max_ or the relative fluorescence × 10^3^.

In order to optimize the reaction conditions, several laboratory experiments were carried out using borate buffer in the range of 7.5-9.5, as Fig. 3 shows, the highest fluorescence reading was obtained upon adding 1mL buffer pH8.5. Concerning the reagent concentration, it was found that the fluorescence intensity increases upon increasing the reagent concentration, but this increase was accompanied by an increase in the background reading, a compromise was obtained using 1 mL of 0.2 μg mL^-1^ solution. Concerning the reaction time, the fluorophore is formed immediately; the fluorescence increases almost linearly by time till reaching a maximum at 30 min (Fig. 4) and remained stable for at least 2 hours at room temperature. Reviewing the literature revealed that PRG has been determined fluorimetrically only through the reaction with NBD-Cl (13); comparing the later reported method with the proposed spectrofluorimetric method exposed that the proposed method using fluorescamine is simpler, as it is operated at room temperature, no need for laborious extraction procedures, while that using NBD-Cl needs heating at 80°C followed by lengthy extraction using chloroform. Moreover it is more sensitive (20-280 ng mL^-1^ compared to 40-400 ng mL^-1^).

### Method II

**Figure 5:**
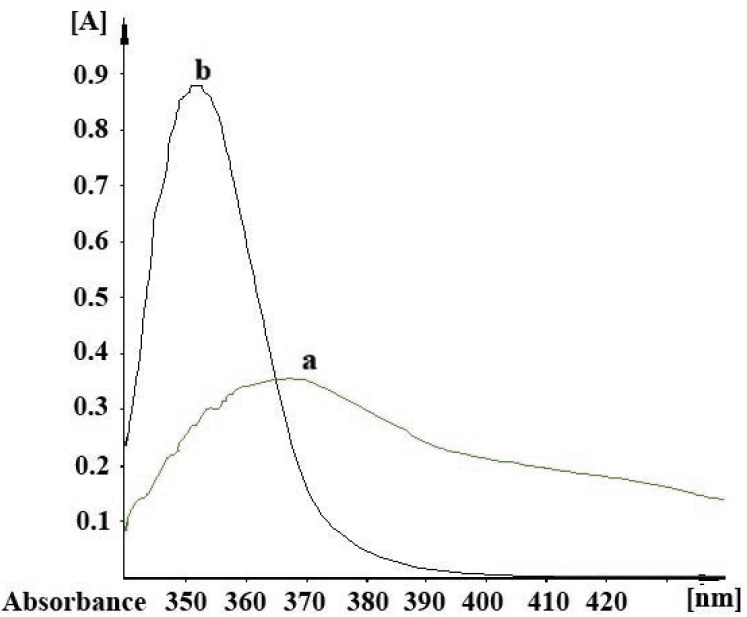
Absorption spectra of the reaction substitution products of PRG: 5 μg mL^-1^ with 2,4- DNFB (a) and 7 μg mL^-1^ with chloranil (b).

**Figure 6:**
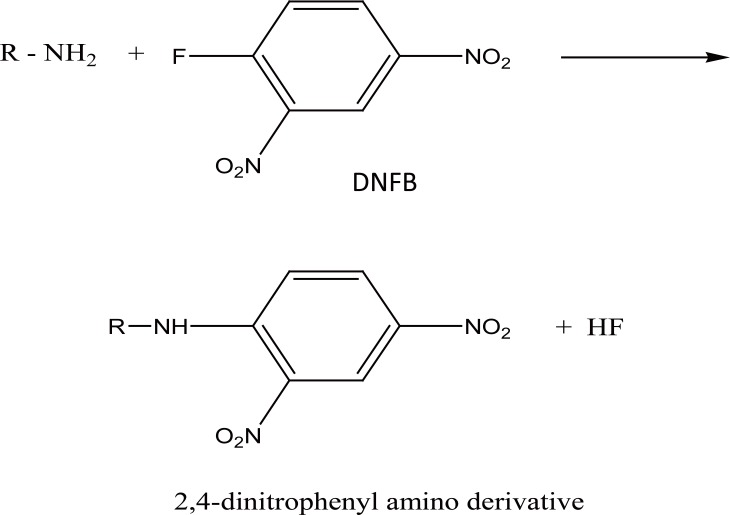
Proposal of the reaction pathway between pregabalin and 2,4- dinitrofluorobenzene.

2,4-Dinitrofluorobenzene (DNFB) has been utilized in pharmaceutical analysis for the determination of specific functional groups such as primary and secondary amines, phenols, thiols and imidazoles (15). In the present study, DNFB reacts through a nucleophilic aromatic substitution reaction with the primary aliphatic amino group in PRG in aqueous alkaline medium to form a yellow colored product (Fig. 5a) as proposed in Figure 6.

The reaction was investigated over the pH range of 7.0-9.4 using borate buffer. The product showed the highest absorption using 1 mL of buffer pH8.0 (Fig. 3). It was found that 0.8 mL of 5 mg mL^-1^ reagent is sufficient to give the maximum color intensity. The excess reagent interference in the absorbance measurement was removed by acid hydrolysis into colorless 2,4-dinitrophenol, 0.1 mL 5 M HCl was enough to accomplish this task. In order to obtain the highest and most stable absorbance, the effect of the reaction time and heating temperature was investigated (Fig. 4). The optimal values are presented in Table 1.

### Method III

**Figure 7:**
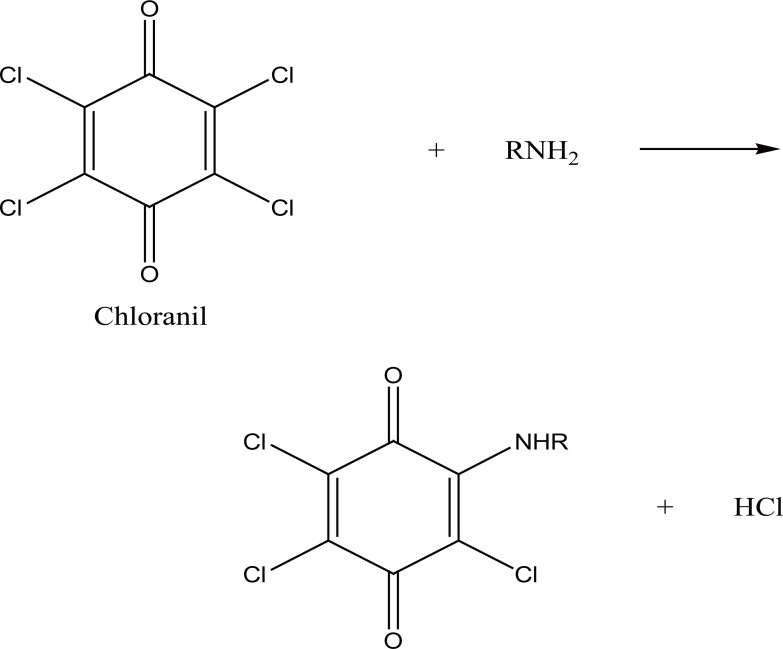
Proposal of the reaction pathway between pregabalin and chloranil.

Chloranil reacts with PRG in alkaline borate buffer to form a yellow colored product exhibiting maximum absorbance at 352 nm (Fig. 5b). The reaction pathway could be explained as a substitution reaction, where the chloro atom is substituted by the amino group in PRG forming the colored chromogen. This was ascertained through testing for the presence of chloride in the medium using silver nitrate in dil. acidic medium. The formation of charge transfer complexes (n→π) is favored in the presence of organic polar solvents (12) and in absence of basic buffers (Figure 7).

Reaction of chloranil with PRG is dependant on the pH of borate buffer, as at pH7 and 7.5, no reaction was developed, and the color starts to develop moderately at pH8 and then increases to a plateau in alkaline pH of 9 and 9.5. The influence of reagent concentration was also investigated carrying the reaction procedure using different concentrations of chloranil in the range of 20-120 μg mL^-1^ (final concentration), It was found that the maximum absorbance was obtained using 3 mL of chloranil (0.246 mg mL^-1^). Concerning the reaction time and heating temperature, the procedure was carried out at 50, 70 and 80°C and the product showed maximum absorbance after heating in a water bath at 70°C for 30 min. The absorbance was stable for at least 2 hours.

The proposed reaction with chloranil in borate buffer medium has several advantages over the reported charge transfer reaction with the same reagent in acetonitrile (12) including higher sensitivity 1-7 μg mL^-1^ compared to 50-400 μg mL^-1^, the proposed method is superior because no organic solvents were used, the concentration of chloranil used is lower than that used in the reported method (0.246 mg mL^-1^ compared to 5 mg mL^-1^), The reaction in alkaline borate buffer is relatively slow and needs heating at 70°C, however, absorbance measurement after few minutes is quite possible but would result in a 30-70% reduction in sensitivity.

### Validation of the proposed methods

**Concentration ranges and calibration graphs.** Under the optimized experimental conditions, the response measured for each method at the specified working wavelengths was found to be proportional to the analyte concentration. The linear regression equations were derived by least-squares treatment of the calibration data. Table 2 summarizes the performance data and statistical parameters for the proposed methods, including concentration ranges, linear regression equations, correlation coefficients, standard deviations of the intercept (S_a_) and slope (S_b_), and SD of residuals (S_y/x_). The high values of the correlation coefficients (>0.999) indicate good linearity over the working concentration ranges.

**Table 2:** Validation data for the determination of pregabalin using the proposed methods

Item	Method		
	I	II	III

Concentration range (μg mL^-1^)	0.02 - 0.28	2 - 7	1 - 7
Regression equation			
Intercept (a)	53.8	- 1.8 × 10^-2^	2.3 × 10^-2^
Slope (b)	2803.12	0.075	0.126
Correlation coefficient (r)	0.9998	0.9996	0.9999
S_a_^a^	4.21	5 × 10^-3^	3 × 10^-3^
S_b_^b^	25.14	1 × 10^-3^	6 × 10^-4^
S_y/x_^c^	6.26	4.56 × 10^-3^	3.29 × 10^-3^
Mean ± SD	99.92 ± 1.15	99.55 ± 1.97	98.30 ± 0.73
RSD%	1.15	1.98	0.74
LOQ^d^ (μg mL^-1^)	3.2 × 10^-3^	1.39	0.6
LOD^e^ (μg mL^-1^)	9.6 × 10^-4^	0.42	0.18

a Standard deviation of the intercept;

b Standard deviation of the slope;

c Standard deviation of residuals;

d Limit of quantitation;

e Limit of detection.

**Precision and accuracy.** The within- and between-day precisions and accuracy were examined by analysis of PRG with the concentrations of 0.04, 0.16 and 0.24 μg mL^-1^ for method I, 2.0, 4.0 and 7.0 μg mL^-1^ for method II and 2.0, 3.0 and 6.0 μg mL^-1^ for method III (n=3). Mean recovery values ± SD, RSD % and Er % were found for all developed methods, and were found to be satisfactory. The obtained results are summarized in Table 3.

**Table 3:** Precision of the proposed methods for analysis of pregabalin

Method	PRG (μg mL^-1^)	Within-day, n=3			Between-day, n=3		
		Mean ± SD	RSD	E_r_%	Mean ± SD	RSD	E_r_%

I	0.04	99.77 ± 1.86	1.86	-0.23	98.58 ± 1.36	1.38	-1.42
	0.16	101.27 ± 0.45	0.44	1.27	101.34 ± 0.46	0.46	1.34
	0.24	98.38 ± 0.60	0.61	-1.62	98.28 ± 0.74	0.76	-1.72
II	2	100.20 ± 0.92	0.91	0.20	100.01 ± 1.21	1.21	0.01
	4	102.31 ± 1.59	1.55	2.31	102.44 ± 1.78	1.74	2.44
	7	99.06 ± 0.81	0.82	-0.94	98.93 ± 0.67	0.68	-1.07
III	2	98.46 ± 1.04	1.05	-1.54	99.90 ± 1.99	1.99	-0.1
	3	99.61 ± 0.86	0.87	-0.39	100.34 ±1.92	1.91	0.34
	6	101.71 ± 1.38	1.36	1.71	98.83 ± 1.32	1.34	-1.17

**Specificity and interference.** Potential interference of the excipients listed by the manufacturer; talc, lactose monohydrate and maize starch in the dosage forms was studied. The results in Table 4 revealed that no interference was encountered from any of these excipients. At the same time and because of the dependence of the reaction in each of the three proposed methods on the presence of a primary aliphatic amino group in the drug molecule, other co-administedred anticonvulsants, anxiolytics and tranquilizers such as chlordiazepoxide, clonazepam, diazepam, nitrazepam and lamotrigine did not interfere. Moreover, synthesis of PRG is mediated through several pathways, as representative examples; the synthesis of PRG from D-mannitol(16) and via quinine-mediated desymmetrization of cyclic 3-isobutylglutaric anhydride (17), where any of these synthesis precursors will not interfere in any of the proposed methods.

**Table 4:** Assay results of pregabalin in its capsules using the proposed methods

Preparation	Method I	Method II		Method III		Reference method (13)

Lyrica^®^ capsules						
% recovery ± SD^a^	99.7 ± 1.122	99.55 ± 1.971		98.30 ± 0.727		98.95 ± 0.869
RSD%^b^	1.125	1.980		0.739		0.878
E_r_ %^c^	-0.3 (t=1.18, F=1.67)	-0.454 (t=0.61, F=5.14)		-1.702 (t=1.18, F=1.43)		-1.046
**ANOVA**						
***Source of Variation***	***SS***	***df***	***MS***	***F***	***P-value***	***F crit***

Between Groups	6.10383055	3	2.03461	1.265415	0.319682	3.238872
Within Groups	25.725756	16	1.60786			
Total	31.8295866	19				

Theoretical values for t and F at *P*=0.05 are 2.31 and 6.39, respectively.

a Mean ± standard deviation for five determinations;

b % Relative standard deviation;

c relative error.

**Detection and quantification limits.** The limits of detection (LOD) and quantification (LOQ) for each proposed method were calculated as 3S/b and 10S/b, respectively, where S is the standard deviation of five blank readings and b is the slope of the corresponding regression equation (Table 2).

**Robustness.** Robustness was examined by evaluating the influence of small variations in different experimental conditions such as working wavelengths, heating temperatures (±2°C) and time (±5 min), borate buffer pH, volume and concentration of reagents. These variations did not have significant effect on the measured responses.

**Stability.** The stability of final measured sample solutions was examined and responses were found to be stable for at least 2 hours at room temperature.

### Applications

**Analysis of capsules.** The obtained satisfactory validation criteria made the proposed methods suitable for the routine quality control analysis of PRG. The proposed methods were successfully applied to the determination of PRG in capsules. The results obtained were statistically compared to the reported method (13) using the student’s t-test for accuracy and the variance ratio F- test for precision as tabulated in Table 4. The experimental values of t and F did not exceed the theoretical values at 95% confidence level; this indicates that there is no significant difference between the compared methods. Single factor analysis of variance (ANOVA) is a powerful tool to compare recoveries obtained from more than two methods (18) and is also used in this work to judge the proposed methods and the calculated F value did not surpass the critical F value. It is evident from the results in Table 4 that all the three proposed methods are applicable to the analysis of PRG in its capsules with comparable analytical performance. Nevertheless, the commendation of a particular method will be based on the experimental conditions such as heating temperature and reaction time or the definitive method sensitivity that determines the amount of specimen required for analysis.

**Application to spiked urine.** The proposed spectrofluorimetric method using fluorescamine was further extended to the *in vitro* determination of PRG in spiked human urine samples. Pregabalin is not bound to plasma proteins and undergoes negligible metabolism. About 98% of a dose is excreted in the urine as unchanged drug (1). In neuropathic patients, PRB is orally given in doses of 150 to 600 mg per day. This concentration fell well within working range of the proposed method (I). The interference resulting from the endogenous amino acids in urine has been corrected by the precipitation of amino acids as their copper salts using copper acetate solution and subsequent filtration of the precipitates (a modification of the Smithers *et al*. method) (14). The results for application of the proposed fluorimetric method to the determination of PRG in human urine samples are summarized in Table 5. These results are satisfactorily accurate and precise.

**Table 5:** Application of the proposed fluorimetric method (I) to the determination of pregabalin in human urine samples

Amount added (μg mL^-1^)	Amount found (μg mL^-1^)	Recovery (%)

0.04	0.0387	96.75
0.04	0.0391	97.75
0.16	0.1552	97.00
0.16	0.1574	98.38
0.2	0.2006	100.30
0.2	0.1956	97.80
Mean ± SD		97.996 ± 1.416
RSD		1.445

## CONCLUSION

The proposed methods can be recommended for routine quality control analysis of PRG where sophisticated equipments are unavailable. The proposed methods are simple, accurate and less tedious than chromatographic procedures. Considering the limits of detection and/or concentrations ranges, the proposed methods are more sensitive than other previously published methods including spectrophotometric (11, 12), and spectroflurimetric (13) methods.

The fluorimetric method is more sensitive, (20-280 ng mL^-1^ compared to 40-400 ng mL^-1^). The absence of interference from added excipients, additives and some co-administered drugs is a noted lead. These advantages encourage the application of the developed method in routine quality control analysis of the drug.

## References

[1] Sweetman SC (2009). Martindale, The complete drug reference.

[2] Ramakrishna N, Vishwottam K, Koteshwara M (2009). Liquid chromatography atmospheric pressure chemical ionization tandem mass spectrometry method for the quantification of pregabalin in human plasma. J. Chromatogr. B.

[3] David B, Millington Ch (2005). Analysis of Pregabalin at Therapeutic Concentrations in Human Plasma/Serum by Reversed-Phase HPLC. Ther. Drug Monit.

[4] Vermeij TAC, Edelbroek PM (2004). Simultaneous high-performance liquid chromatographic analysis of pregabalin, gabapentin and vigabatrin in human serum by precolumn derivatization with o-phtaldialdehyde and fluorescence detection. J. Chromatogr. B.

[5] Douša M, Gibala P, Lemr K Liquid chromatographic separation of pregabalin and its possible impurities with fluorescence detection after postcolumn derivatization with o-phtaldialdehyde. J. Pharm. Biomed. Anal.

[6] Liang Ch, Guohai W, Jianzheng Ch (2009). Enantiospecific analysis of pregabalin by HPLC-ELSD. Fujian Fenxi Ceshi.

[7] Jadhav AS, Pathare DB, Shingare MS (2007). Validated enantioselective LC method, with precolumn derivatization with Marfey’s reagent, for analysis of the antiepileptic drug pregabalin in bulk drug samples. Chromatographia.

[8] Xiaohui Ch, Daolin Z, Jie D (2008). Determination of optical impurity of pregabalin by HPLC with pre-column chiral derivatization. J. Chromatogr. Sci.

[9] Béni S, Sohajda T, Neumajer G (2010). Separation and characterization of modified pregabalins in terms of cyclodextrin complexation, using capillary electrophoresis and nuclear magnetic resonance. J. Pharm. Biomed. Anal.

[10] Rajinder Singh G, Manirul H SK, Prem Sh (2009). Development and validation of pregabalin in bulk, pharmaceutical formulations and in human urine samples by UV spectrophotometry. IJBS.

[11] Onal A (2009). Development and validation of selective spectrophotometric methods for the determination of pregabalin in pharmaceutical preparation. Chinese J. Chem.

[12] Salem H (2009). Analytical study for the charge-transfer complexes of pregabalin. E- J. Chem.

[13] Onal A, Olcay S (2009). Spectrophotometric and spectrofluorimetric methods for the determination of pregabalin in bulk and pharmaceutical preparation. Spectrochim. Acta A.

[14] Belal F, Abdine H, Al-Majed A (2002). Spectrofluorimetric determination of vigabatrin and gabapentin in urine and dosage forms through derivatization with fluorescamine. J. Pharm. Biomed. Anal.

[15] Conors KA (1973). Reaction mechanisms in organic analytical chemistry.

[16] Izquierdo S, Aguilera J, Buschmann HH (2008). Stereoselective and efficient synthesis of (*S*)-pregabalin from d-mannitol. Tetrahedron: Asymmetry.

[17] Hamersak Z, Stipetic I, Avdagic A (2007). An efficient synthesis of (*S*)-3-aminomethyl-5-methylhexanoic acid (Pregabalin) via quinine-mediated desymmetrization of cyclic anhydride. Tetrahedron: Asymmetry.

[18] Miller JN, Miller JC (2000). Statistics and Chemometrics for Analytical Chemistry.

